# Recent increase in asthma mortality in Brazil: a warning sign for the public health system

**DOI:** 10.36416/1806-3756/e20240138

**Published:** 2024-11-16

**Authors:** Marcos Brum, Jordana Henz, Mariana Boeira, Simoni Soares, Frederico Friedrich, Paulo Márcio Pitrez

**Affiliations:** 1. Escola de Medicina, Pontifícia Universidade Católica do Rio Grande do Sul - PUCRS - Porto Alegre (RS) Brasil.; 2. Santa Casa de Misericórdia de Porto Alegre, Pavilhão Pereira Filho, Porto Alegre (RS) Brasil.

**Keywords:** Public health, Asthma, Mortality

## Abstract

**Objective:**

To provide an update on asthma mortality trends in Brazil and its regions between 2014 and 2021.

**Methods:**

This was a retrospective descriptive observational study based on asthma mortality data from the Brazilian National Ministry of Health Mortality Database for the 2014-2021 period.

**Results:**

In the study period, there were 18,584 asthma deaths in Brazil, with an annual increase of 2.5%, corresponding to 0.03 deaths/100,000 population (95% CI, 0.01-0.04; p = 0.01). The northeastern region of the country had the highest prevalence of asthma deaths (1.50 deaths/100,000 population), and the southern region showed the greatest variation in the study period (44%). We observed a higher proportion of deaths among females and elderly patients, and when analyzing asthma deaths by place of occurrence, we observed that 28% of all deaths occurred at home.

**Conclusions:**

Asthma mortality remains high and shows an increasing trend for the first time in the past decades. This constitutes an important public health concern, given the treatable nature of the disease.

## INTRODUCTION

Asthma is one of the most common noncommunicable diseases, with a substantial impact on the quality of life of patients.^1^ Asthma is considered a public health issue in Brazil and worldwide, and its prevalence has been rising in the past decades, demanding closer attention from health authorities and the medical community.^2^ ^,^ ^3^ Epidemiological data reveal the extent of this challenge, raising the need for in-depth studies to understand the underlying factors and develop more effective strategies of prevention and treatment.^4^ The prevalence of asthma has been shown to be high in many countries, including those in Latin America.^2^ Asthma mortality is an important outcome to be monitored by public health systems worldwide.^3^ ^,^ ^5^

In Brazil, until 2013, studies based on data from the *Departamento de Informática do Sistema Único de Saúde* (DATASUS, Information Technology Department of the Brazilian Unified Health Care System) and the *Instituto Brasileiro de Geografia e Estatística* (IBGE, Brazilian Institute of Geography and Statistics) showed a historical reduction in asthma mortality.^6^ By updating asthma mortality data in Brazil we are able to understand the dynamics of the disease and the factors that influence it; this is essential to guide public health policies and appropriate interventions aimed at improving the quality of life of asthma patients and reducing the burden of asthma morbidity and mortality.^7^ The availability of open data from death certificates and ICD-10 codes allows us to perform a detailed evaluation of the trends in asthma mortality in Brazil.^8^ The objective of the present study was to provide an update on asthma mortality trends in Brazil and its regions between 2014 and 2021. 

## METHODS

This was a retrospective descriptive observational study based on asthma mortality data from the *Sistema de Informações Sobre Mortalidade do Ministério da Saúde do Brasil* (SIM, Brazilian National Ministry of Health Mortality Database), available at https://opendatasus.saude.gov.br/, for the 2014-2021 period.^9^ The study period was defined on the basis of the fact that a previous report covers the 2008-2013 period.^6^ The study population consisted of all asthma deaths (ICD-10 codes J45 and J46) among individuals > 6 years of age.^7^ Deaths from COVID-19 alone (ICD-10 codes B34.2 and U07.2) were not included in the analysis. 

We also conducted a geographic analysis, correcting the variables for the population size as assessed by the 2022 IBGE census and selecting the population > 6 years of age. The extracted data were subdivided into Brazil and its regions (central-west, north, northeast, south, and southeast). To analyze asthma deaths exclusively, we used the SIM variable “*causabas*,” which refers to the underlying cause of death. According to the SIM Procedures Manual, the underlying cause of death is understood as the disease or injury that started the succession of morbid events that directly led to death.^10^

The mortality rate was calculated by dividing the total number of asthma deaths (stratified by country region, age group, sex, and place of occurrence) by the corresponding population (within each stratification), multiplied by 100,000.^11^ The relative variation for each year was calculated in relation to the year 2014. To obtain the mean percentage of variation for each stratum, we calculated the average of the variations. 

To compare prevalence rates, we employed a linear regression model. To compare proportions, we used the chi-square test. To analyze the number of years of schooling, we selected adults > 18 years of age. The significance level was set at 5%. The data were processed with R studio, version 2023.6.01 (The R Foundation for Statistical Computing, Vienna, Austria). The SIM is in the public domain, requiring no research ethics committee approval. The analysis script (in R language) is available at https://github.com/mobrant94/asthma_mortality. 

## RESULTS

**Figure 1:**
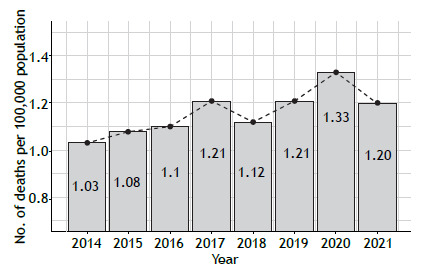
Overall mortality from asthma in Brazil between 2014 and 2021.

**Figure 2:**
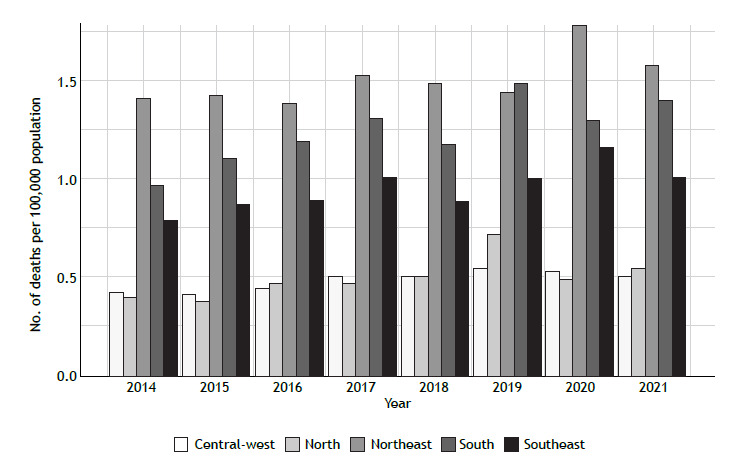
Asthma mortality in Brazil, by region.

Between 2014 and 2021, 18,584 asthma deaths were recorded in Brazil (Figure 1), with a mean prevalence of 1.16/100,000 population per year. There was an increase of 14% in asthma deaths, corresponding to 0.03 deaths/100,000 population per year (95% CI, 0.01-0.04; p = 0.01). The northeastern region showed the highest annual average (of 1.50 deaths/100,000 population), whereas the central-western region had the lowest annual average (of 0.48 deaths/100,000 population). The southern region showed the greatest variation during the study period, from 0.97 deaths/100,000 population in 2014 to 1.39 deaths/100,000 population in 2021, corresponding to an increase of 44%, which is equivalent to 0.06 deaths/100,000 population per year (95% CI, 0.01-0.09; p < 0.01). On the other hand, the northeastern region showed the lowest variation, with 1.41 deaths/100,000 population in 2014 and 1.58 deaths/100,000 population in 2021, an overall increase of 12%, corresponding to approximately 0.03 deaths/100,000 population per year (95% CI, 0.01-0.07; p = 0.01; Figure 2). 

**Figure 3:**
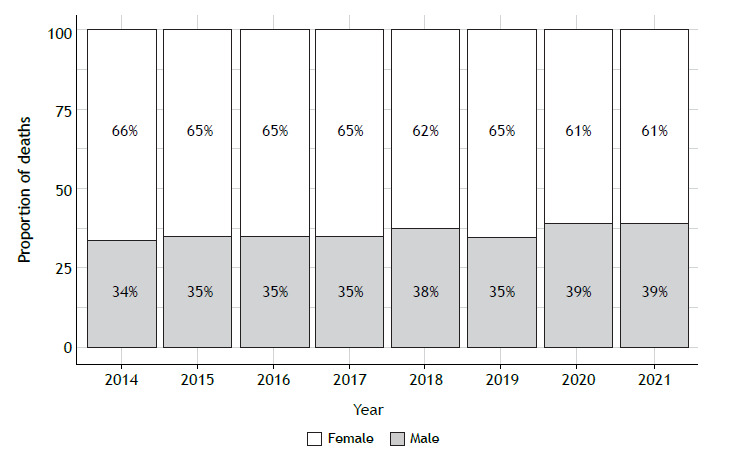
Proportion of asthma deaths in Brazil, by sex.

**Figure 4:**
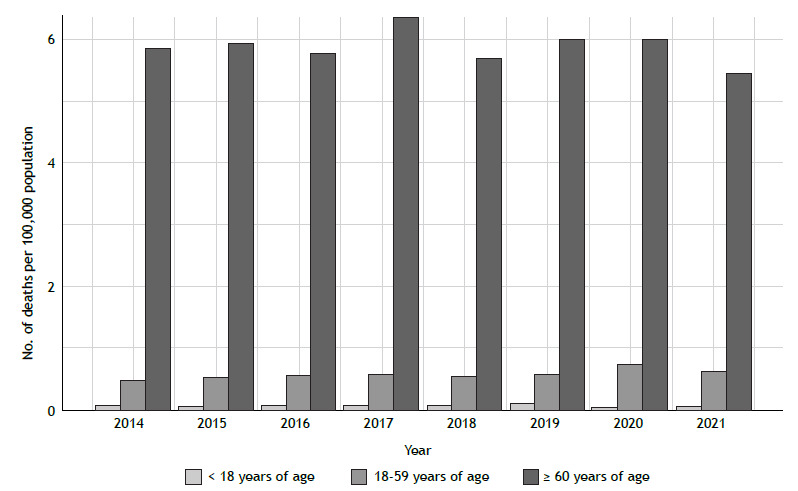
Asthma mortality in Brazil, by age group.

The highest proportion of deaths was observed in females (Figure 3), accounting for an annual average of 64% of total deaths (p < 0.01). In the population ≥ 60 years of age, we observed an asthma mortality prevalence that was 10 times higher than that in the 18- to 59-year age bracket (5.87 vs. 0.59 deaths/100,000 population). As for the variation in each age group, individuals < 18 years of age showed an overall reduction of 10% and an annual decrease of 0.01 deaths/100,000 population (95% CI, −0.01 to 0.01; p = 0.88). In the 18- to 59-year age bracket, a 19% increase was observed, with an annual increase of 0.02 deaths/100,000 population (95% CI, 0.01-0.04; p = 0.03). In the group of individuals ≥ 60 years of age, there was an overall reduction of 0.5% and an annual decrease of 0.03 deaths/100,000 population (95% CI, −0.13 to 0.07; p = 0.47). Proportionally, the group of individuals < 18 years of age accounted for 2% of all deaths in the study period, followed by the group of individuals in the 18- to 59-year age bracket (30%) and that of those ≥ 60 years of age, which accounted for 68% of all asthma deaths (p < 0.01; Figure 4).

**Figure 5:**
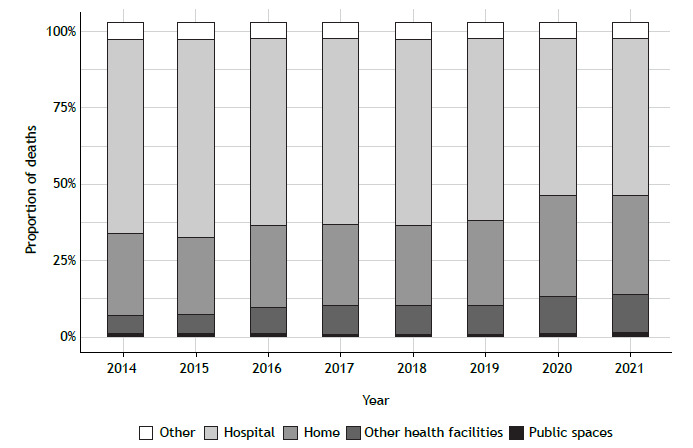
Proportion of asthma deaths in Brazil, by place of occurrence.

Regarding the proportion of deaths by place of occurrence (Figure 5), 59% occurred at a hospital, 28% occurred at home, 10% occurred in health facilities other than hospitals, and 3% occurred in the street or in public spaces (p < 0.01). Between 2014 and 2021, deaths occurring in health facilities other than hospitals or at home showed average increases of 86% and 23%, respectively. Additionally, we observed a significant increasing trend in both categories (p < 0.05). 

**Figure 6:**
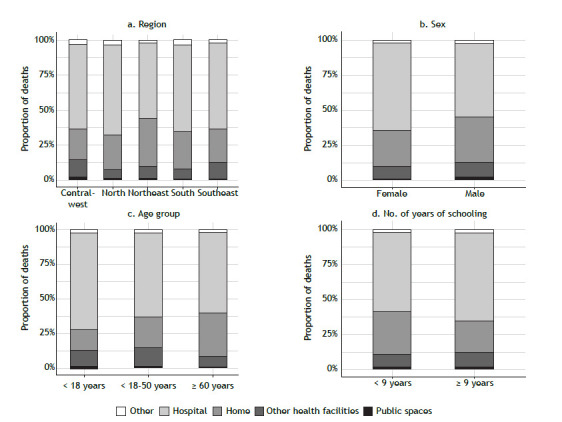
Proportion of asthma deaths in Brazil, by place of occurrence, in relation to region, sex, age group, and number of years of schooling.

Regarding deaths by place of occurrence and region (Figure 6a), the northeastern region showed the highest proportion of deaths occurring at home (35%), whereas the northern region had the lowest proportion of deaths occurring at home (22%). Regarding sex (Figure 6b), the proportion of deaths occurring at home was higher among males than among females (32% vs. 26%). When looking at the age groups (Figure 6c), we found that the group of individuals < 18 years of age accounted for 15% of all deaths occurring at home, whereas that of those ≥ 60 years of age accounted for 31% of all deaths occurring at home. Regarding the level of education (Figure 6d), the proportion of deaths occurring at home was higher among individuals who had had < 9 years of schooling than among those who had had ≥ 9 years of schooling (30% vs. 22%). 

## DISCUSSION

We found an increase of 14% in asthma mortality in Brazil between 2014 and 2021. Asthma mortality in Brazil remains high despite regional disparities, with an average of 6 asthma deaths/day. The group of individuals ≥ 60 years of age showed the highest mortality rate, and females showed the highest proportion. The proportion of deaths that occurred at home in Brazil was 28%. 

Studies conducted before 2015 showed a progressive decrease in the number of asthma deaths in Brazil, showing a reduction of 46.2% between 1980 and 2014, and of 10% between 2008 and 2013.^6^ ^,^ ^12^ A decreasing mortality trend was also observed between 1980 and 2010, with an annual percentage reduction of approximately 2%.^7^ ^,^ ^13^ Global data also showed a downward trend in asthma mortality, with a 57% decrease in global mortality between 1993 and 2006, and an approximate mortality rate of 0.9/100,000 population from 2006 to 2012.^14^ However, our study showed an increase of asthma deaths of approximately 2.5% per year from 2014 to 2021. Estimates from the WHO corroborate our findings, showing an increasing trend in asthma mortality rates between 2014 and 2020 in Brazil, by approximately 30% (1 vs. 1.3/100,000 population).^15^

In the present study, we observed a peak in mortality in 2020, the first year of the COVID-19 pandemic. Although we excluded deaths directly related to COVID-19, this increase may reflect the indirect impacts of the pandemic, such as reduced access to health care and poor diagnosis of respiratory conditions. Furthermore, although the SIM/DATASUS is robust, it is subject to underreporting and misclassification. Our findings highlight the need for greater accuracy in death records and the management of respiratory diseases during pandemics. 

We found higher asthma mortality rates in the northeastern region of Brazil. Data published between 2008 and 2013 showed that the southeastern and southern regions had higher in-hospital asthma mortality rates.^6^ ^,^ ^16^ On the other hand, studies evaluating overall mortality between 1998 and 2009, and in individuals ≤ 19 years of age between 1996 and 2015 in Brazil showed that the northeastern region had higher overall asthma mortality rates.^17^ ^,^ ^18^ Although we did not directly assess the Human Development Index, it is important to consider that the northeastern region of Brazil historically has one of the lowest Human Development Indices, which could be indicative of disparities in access to health care and the quality of medical care.^19^

Asthma-related mortality was found to be higher in females than in males in the present study, with asthma deaths among females accounting for 64% of all asthma deaths. This finding is consistent with data from the United States.^20^ However, we observed a significant increasing trend in mortality among males, which is consistent with previous studies.^15^ ^,^ ^21^ Further studies are needed to find out why females are at a higher risk of death from asthma. 

Individuals ≥ 60 years of age showed the highest asthma mortality rates in the study period. Previous studies have also found higher asthma mortality rates in the elderly.^7^ ^,^ ^22^ ^,^ ^23^ Data from the Centers for Disease Control and Prevention for the 2006-2018 period in the United States showed a higher mortality rate among individuals > 65 years of age (29.5 per million) and a lower mortality rate among younger individuals, with a 1.6 per million rate in children in the 0- to 4-year age bracket.^24^ One Brazilian study, which also used data from the DATASUS (for the 1980-2012 period), found a higher mortality rate in individuals > 75 years of age, as well as an increase in the proportion of asthma deaths in older age groups over time.^7^ We acknowledge that elderly patients may have a higher asthma mortality rate because of the presence of comorbidities, highlighting the need for a protocol for the clinical management of severe asthma in elderly patients in the public health system. 

The place of death occurrence is an important aspect to be considered when developing public health strategies to reduce asthma mortality. Deaths occurring at home or in a hospital setting can influence prevention strategies for asthma-related deaths by public health officials. Our study showed that approximately one third of all asthma deaths occurred at home, and the northeastern region had the highest proportion of at-home deaths (35%). In Denmark, one study showed that approximately 25% of all individuals who had a sudden death at home had asthma.^25^ Regarding age groups, we showed that 15% and 31% of all at-home deaths occurred in individuals < 18 years of age and ≥ 60 years of age, respectively. The high prevalence of at-home asthma deaths in our study shows that this must be a priority concern for the public health system. Furthermore, we found that individuals who had had < 9 years of schooling had a higher proportion of deaths at home than did those who had had ≥ 9 years of schooling. Studies suggest that lower parental or individual educational levels are risk factors for mortality.^18^ Factors such as region, sex, age, and educational level do not seem to be associated with the place of death occurrence. 

Our study has some limitations. First, because our study involves secondary data, there is a risk of issues related to cause of death notification, such as underreporting of asthma. However, previous studies have shown that mortality records in Brazil have a data completeness of 98%.^26^ Another limitation refers to the input of DATASUS data. To minimize this and ensure that the data were treated reliably, we collected and analyzed data consolidated in the SIM 12 months after the index year. On the basis of our previous experience using the DATASUS, a period of 12 months is enough for the database to present the final figures.^27^ The COVID-19 pandemic period included in the study may introduce some bias, particularly regarding the difficulty in accessing health care services for non-COVID-19 conditions, which could have influenced asthma mortality patterns during the study period. Finally, these limitations are mitigated by the fact that our update on asthma mortality trends is based on official data from the Brazilian National Ministry of Health.^28^

In conclusion, asthma mortality remains high and shows an increasing trend for the first time in the past decades. This constitutes a serious public health issue, given the treatable nature of the disease. Our findings highlight the need for greater attention to asthma diagnosis and management in Brazil, particularly in the primary health care setting. 
